# Characterizing the Influence of Relative Humidity and Ethanol Content on the Dynamic Size Distributions of Aerosols Generated from a Soft Mist Inhaler

**DOI:** 10.1007/s11095-025-03851-1

**Published:** 2025-04-01

**Authors:** Yiliang Lance Jiang, Jose R. Ruiz, Richard Friend, Jonathan P. Reid

**Affiliations:** 1 University of Bristol, School of Chemistry, Cantock’s Cl, Bristol, BS8 1TS UK; 2 Chiesi Ltd., 1 Bath Road Industrial Estate, Bath Rd, Chippenham, SN14 0AB UK

**Keywords:** electrodynamic balance, ethanol content, relative humidity, soft mist inhaler, comparative hygroscopic aerosol particle sizing

## Abstract

**Objective:**

Inhaled drug delivery systems need to ensure that the delivered aerosol effectively reach the lungs while overcoming challenges related to environmental conditions, such as relative humidity (RH). This study investigates the impact of environmental factors on aqueous aerosol behaviour using a Respimat® Soft Mist Inhaler (SMI) formulated with and without ethanol content.

**Methods:**

Comparative Hygroscopic Aerosol Particle Sizing (CHAPS) was used to measure aerosol size distribution under varying RH levels, while single droplet analysis was conducted using Comparative Kinetics-Electrodynamic Balance (CK-EDB) to assess particle behaviour.

**Results:**

The findings reveal that increased RH results in larger particle sizes, while elevated ethanol content consistently decreases both particle size and mass. The strong agreement between CHAPS measurements and CK-EDB data suggests that aerosol plume behaviour can be accurately modelled from single droplet data.

**Conclusion:**

The study highlights ethanol's role in optimizing particle size distribution, which is crucial for enhancing the therapeutic efficiency of inhaled medications. These results underscore the importance of tailoring formulation and environmental conditions to improve drug delivery outcomes in pulmonary therapies and the importance of recognising that aerosol particle size distributions are dynamic and highly compositionally dependent.

**Supplementary Information:**

The online version contains supplementary material available at 10.1007/s11095-025-03851-1.

## Introduction

Pulmonary delivery provides a large surface area for therapeutic aerosols delivered by inhalation, facilitating both local and systemic therapies while bypassing first-pass metabolism [1, 2]. Factors such as mucociliary clearance, airway geometry and humidity contribute towards the maintenance of the sterility of the lung, but also act as obstacles to the effectiveness of inhaled medications [1]. Particles of 1–5 μm diameter are often considered as the ideal size for reaching the small airways [3, 4]. To generate particles within this size range, various devices have been developed over the years. Aqueous solutions are usually actuated using either a nebulizer or a soft mist inhaler (SMI). Nebulizer treatments typically take minutes to complete, while SMI treatment may only take one or two breaths [5]. SMIs are portable and generally require less maintenance compared to nebulizers, making them an increasingly popular device for the delivery of inhaled therapeutic aerosols.

The relative humidity (RH) can play a crucial role in the efficacy of inhaled medicines. Legh-Land *et al*. [6] has determined that the water activity of a pMDI droplet at the point of inspiration can be as high as ~ 0.98, which is equivalent to an equilibrium moisture content at 98% RH. For aqueous solutions used in SMIs, the water activity is usually above 98% at actuation based on the mass fraction of the solute in solution. The ambient air is usually within the range 40 – 60% RH and the RH within the respiratory system can rise up to above 99% RH in the alveoli region to ensure efficient gas exchange and prevent delicate tissues from drying out [7]. Therefore, depending on the inhalation device and accessories used (e.g., holding chambers), and the physiological state of the patient’s respiratory system, the aqueous therapeutic particles can experience periods of drying and re-humidification following aerosolization, during inhalation and through to deposition. Understanding how the hygroscopicity of aerosols affects droplet size, and thus the deposition pattern within the respiratory tract, is crucial for predicting the therapeutic dose that will be delivered to the target site [6, 8].

To achieve the desired therapeutic effect and to enhance the shelf life of the active ingredient, various excipients are often included in inhaled medications. Ethanol is commonly utilized to enhance solubility of the active pharmaceutical ingredient, stability and aerosolization of the formulation, and inhibit microbial growth [9, 10]. Its presence can influence aerosol properties, such as the droplet size distribution on generation and droplet evaporation rate as ethanol volatilises, in turn affecting the drug deposition profile and the efficiency of active ingredients [11]. Understanding the impact of ethanol on the size distribution of therapeutic sprays is crucial for optimizing the formulation development of inhaled medications.

In this study, we examine the influence of environmental RH on the size distribution of saline aerosol droplets in a plume generated by the Respimat® Soft Mist Inhaler (SMI) device. We utilize the Comparative Hygroscopic Aerosol Particle Sizing (CHAPS) method and compare the results with those obtained from the comparative kinetics-electrodynamic balance (CK-EDB) [12], a single droplet characterization method. Both methods will be introduced in the next section. If the plume and single particles respond similarly to changes in RH, we can develop models based on single particle data to predict the evolving size distribution of the aerosol plume. The results of the comparison between CK-EDB and CHAPS output on varied RH conditions are discussed in Section "Influence of Relative Humidity on the Size Distribution of Aqueous Solute Aerosol". We also investigate the impact of initial ethanol content in the formulation of mannitol and salbutamol sulphate on particle size distribution using CHAPS under different RH conditions in Section "Influence of Ethanol on Aerosol Size Distribution".

## Methods

### Inhaler Device and Formulations

The inhaler device used in this study is a Respimat® SMI (Boehringer Ingelheim International GmBH, Ingelheim am Rhein, Germany). Ingredients used in this study include: sodium chloride (> = 99.5% purity, Fisher Scientific, United Kingdom), mannitol (98 + % purity, Fisher Scientific, United Kingdom), water (HPLC grade, VWR Chemicals, France), ethanol (HPLC grade, > 99.8% purity, Sigma-Aldrich, United Kingdom) and salbutamol sulphate, which was provided courtesy of Chiesi Limited (Chippenham, UK). In addition to the Respimat® SMI, an Omron nebulizer was used for comparison in select experiments to assess consistency in aerosol hygroscopicity across different atomization methods.

To provide a structured overview of the different formulations tested in this study, Table I summarizes the compositions used and the devices employed for aerosol generation. Sodium chloride (NaCl) is commonly used as a reference hygroscopic aerosol in inhalation studies [13], while mannitol is utilized in dry powder inhalers for lung function assessment due to its hygroscopic properties [14]. Salbutamol sulphate, a widely used bronchodilator, serves as a model drug formulation for evaluating aerosolized medication performance in treating respiratory conditions such as asthma and COPD [15].

**Table I:** Summary of Formulations and Device Usage

Formulation	Purpose	Device Used
3% w/w NaCl in water	Evaluate hygroscopic growth	Respimat® SMI, Omron Nebulizer
3% w/w Mannitol in water	Evaluate hygroscopic growth	Respimat® SMI
2% w/w Mannitol + Ethanol (0–75%)	Effect of ethanol on evaporation kinetics	Respimat® SMI
2% w/w Salbutamol Sulphate + Ethanol (0–75%)	Effect of ethanol on evaporation kinetics	Respimat® SMI

### Plume Characterization: Comparative Hygroscopic Aerosol Particle Sizing (CHAPS)

**Fig. 1:**
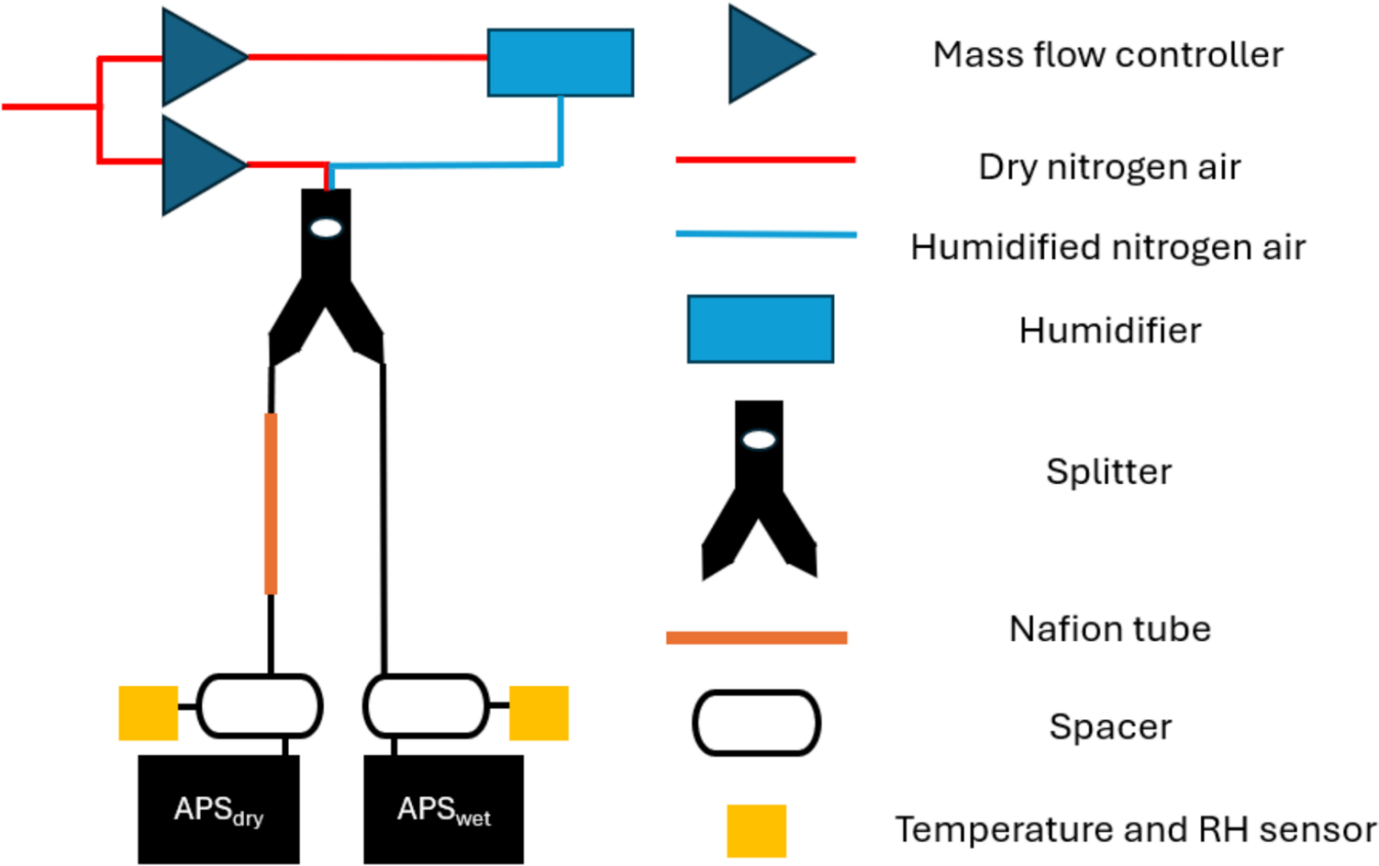
Diagram illustrating the CHAPS set-up

A detailed description of the CHAPS method has been presented recently, and only a brief overview is provided in this section. As shown in Fig. 1, CHAPS is based around the implementation of two aerodynamic particle sizers (Model 3221, TSI) working in parallel to measure aerosol size distribution at two different humidity levels from the same plume. The RH of the flow system is controlled upstream of the aerosol introduction point by adjusting the balance in a mixture of dry and humidified nitrogen gas using two mass flow controllers (Model: F-201CV; Bronkhorst, UK). The room temperature is maintained at 20 ± 1°C using an air conditioning unit. While temperature influences evaporation kinetics and mass transport, it does not affect equilibrium state measurements such as equilibrated particle size growth factors, which are primarily governed by RH and solute composition.

The dry nitrogen gas (0% RH) is sourced from the university’s centralized gas supply system (boil-off from a liquid nitrogen supply) and is humidified by passing through a bubbler (humidifier) filled with deionized water (Fig. 1). The RH in APS_wet_ flow can be reliably maintained at 95 – 98% RH after equilibrating. A lower humidity condition is achieved in one of the CHAPS flow branches by passing the aerosol flow through a Nafion dryer (Model: MD-700-24S-3, Perma Pure) prior to size measurement with the APS_dry_. Particle size distributions, ranging from 0.5 μm to 20 μm, are monitored at a temporal resolution of 1 sample per second from both APS devices. The data are processed using Aerosol Instrument Manager software (version 10.3, TSI), and a bimodal distribution is fitted to the dataset using a custom Python script.

The flow splitter and two spacers, one installed in the flow prior to each APS inlet, are 3D printed using a Raise3D Pro2 3D printer (Raise3D, USA). Polylactic acid (PLA) filament (1.75 mm diameter, RS PRO, Netherlands) is selected for its water resistance, making it ideal for high-humidity environments. Alternative materials can be used for 3D printing the spacer if more chemically aggressive aerosols are involved. Black filament is used in case photosensitive ingredients need to be tested.

### Single Particle Characterization and Prediction

#### Comparative Kinetics – Electrodynamic Balance (CK-EDB)

The CK-EDB is used to determine the physicochemical properties of single aerosol droplets experimentally. Although measurements are made on droplets with an initial radius of ~30 μm, the CK-EDB data provide a rigorous test of thermodynamic and kinetic models of droplet behaviour allowing robust predictions of the behaviour of smaller, respirable droplets. This method, detailed by Rovelli *et al*. [16], allows control of temperature and RH of the vapour surrounding the trapped particle by adjusting a mix of dry and humidified nitrogen flow using mass flow controllers. The temperature is managed by circulating ethylene glycol coolant through the electrode assembly.

A droplet-on-demand generator ejects charged droplets into the CK-EDB trapping chamber, where they are levitated by an electrodynamic field. A 532 nm laser beam illuminates the droplet, and a CCD camera collects the elastic scattering. From the scattering pattern, often referred to as the phase function, the droplet size can be estimated. Mie theory is applied to estimate droplet size, requiring precise parameterisations of droplet density and refractive index obtained with change in water content. Compositionally dependent parameterisations are obtained from bulk phase solution measurements using a Densito meter and a Palm Abbe refractometer, respectively, up to the bulk solubility limit with robust and previously verified extrapolations made into supersaturated solution conditions. These parameterisations are integrated into the CK-EDB data output and analysis for accurate predictions of droplet hygroscopicity using previously verified methods [17].

A second DoD generator produces a probe droplet with well-characterized thermodynamic and kinetic properties, which is used to estimate the RH within the chamber. The hygroscopic response of aerosol sample droplets is assessed by measuring the particle size during evaporation and equilibrium at varying RH levels. Droplet size in the CK-EDB is reported as the optical diameter, which is assumed to be equivalent to the geometric diameter for spherical particles.

#### Density Corrections for Solution Properties Estimated from Thermodynamic Models

The Extended Aerosol Inorganics Model (E-AIM) is a widely used to predict the thermodynamic properties of inorganic salts, such as sodium chloride, under varying humidity conditions [18–20]. It has demonstrated a high degree of agreement with the experimentally measured hygroscopic growth curves for common salts using the EDB [21]. For more complex organic compounds, such as mannitol and salbutamol sulphate, the CK-EDB technique is employed to measure the radial growth factor (rGF) curve. Both E-AIM and CK-EDB determine the particle size as volume-equivalent diameters. However, to compare with the CHAPS data, which provides aerodynamic diameters, a conversion is required, as shown in Eq. 1:

1 $${D}_{ae}={D}_{ve}\sqrt{\frac{1}{\chi }\frac{{\rho }_{p}}{{\rho }_{0}}\frac{{C}_{c}({D}_{ve})}{{C}_{c}\left({D}_{ae}\right)}}$$

where ${D}_{ae}$ is the aerodynamic diameter, ${D}_{ve}$ is the volume-equivalent diameter, ${\rho }_{p}$ is the particle density, ${\rho }_{0}$ is the reference density (1 g/cm^3^), $\chi$ is the shape factor, and $\frac{{C}_{c}({D}_{ve})}{{C}_{c}\left({D}_{ae}\right)}$ represents the ratio of the Cunningham slip correction factor between the volume equivalent and aerodynamic diameters.

When aerosol particles are actuated from an inhaler device in an aqueous droplet form and remains in conditions above the efflorescence RH (the point at which crystallization occurs), they can be assumed to maintain a spherical shape ($\chi$=1). Furthermore, since the optimal aerodynamic diameter for therapeutic particles is within the 1 – 5 μm range, balancing the inertia force and Brownian motion [22, 23], the Cunningham slip correction factor can be neglected. This correction becomes significant only for particles in the nanoscale range. Hence, for this study, only the particle density is required for the conversion from ${D}_{ve}$ to ${D}_{ae}$. As described above, density parameterizations can be obtained using a Densito density meter (Mettler Toledo, UK), providing the relationship between particle density and the solute mass fraction (MFS).

The Aerosol Inorganic–Organic Mixtures Functional groups Activity Coefficients (AIOMFAC) model enables the prediction of the thermodynamic properties of aqueous solutions of organic compounds, such as mannitol. The model output includes the correlation between MFS and water activity, which is equivalent to RH at equilibrium [24–26]. By combining the MFS *vs*. RH relationship from AIOMFAC with the particle density *vs*. MFS data from the density parameterization using Densito, the water activity *vs*. density correlation can be derived. This correlation is then used for converting ${D}_{ve}$ to ${D}_{ae}$ across a range of RH.

## Results

### Influence of Relative Humidity on the Size Distribution of Aqueous Solute Aerosol

The rGF curve of sodium chloride (NaCl), derived from E-AIM data, is reported in Figure S1. The rGF reflects the degree to which a particle grows at a given equilibrated RH compared to its size at RH = 0%. Since E-AIM yields the geometric rGF (rGF_geo_), the conversion to aerodynamic rGF (rGF_aero_) necessitates applying density parameterization and the correlation between MFS and water activity from the E-AIM model. In the lower RH range, the rGF_aero_ curve displays a flatter slope, with higher rGF values, a consequence of the increased density of the particle. As the RH exceeds 90%, the geometric and aerodynamic rGF curves converge due to the increased water content in the aerosolized droplet, which causes the density to approach that of water (~ 1 g/cm^3^) at higher RH levels. Thus, the influence of a density correction on rGF_geo_ to estimate rGF_aero_ becomes minimal under the high RH conditions.

**Fig. 2:**
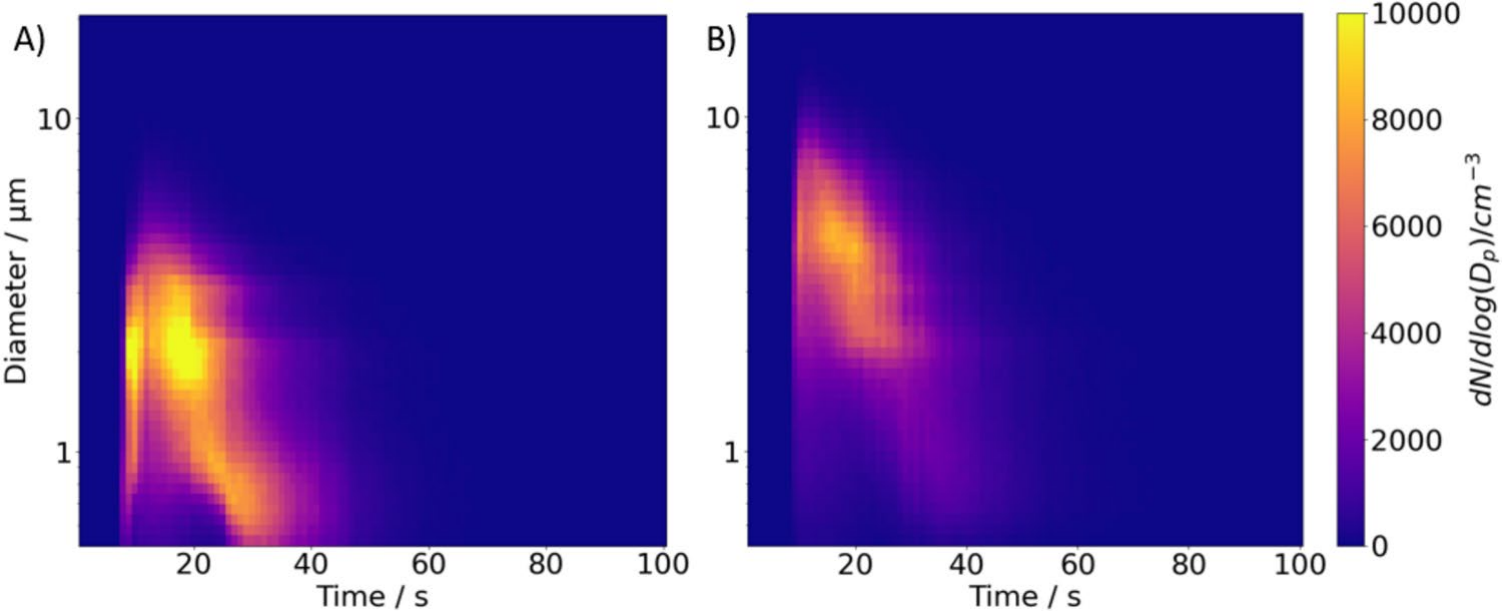
Plume development of 3% w/w NaCl actuated from the Respimat® SMI at 20°C: (**A**) 60% RH in APS_dry_, and (**B**) 94% RH in APS_wet_. The colour represents the particle number concentration.

The concurrent CHAPS measurements enable an analysis of plume development over time under different RH levels. At the start of data recording (time = 0), approximately 10 s of background particle concentration is recorded to ensure baseline uniformity before actuation. Figure 2 provides examples of the evolving size distributions, with measurements taken at 1-s intervals. At 60% RH (Fig. 2A), the particle diameters are noticeably smaller compared to those at 94% RH (Fig. 2B). The colour intensity in Fig. 2 indicates the particle number concentration, highlighting the variations in particle size-distribution over time.

**Fig. 3:**
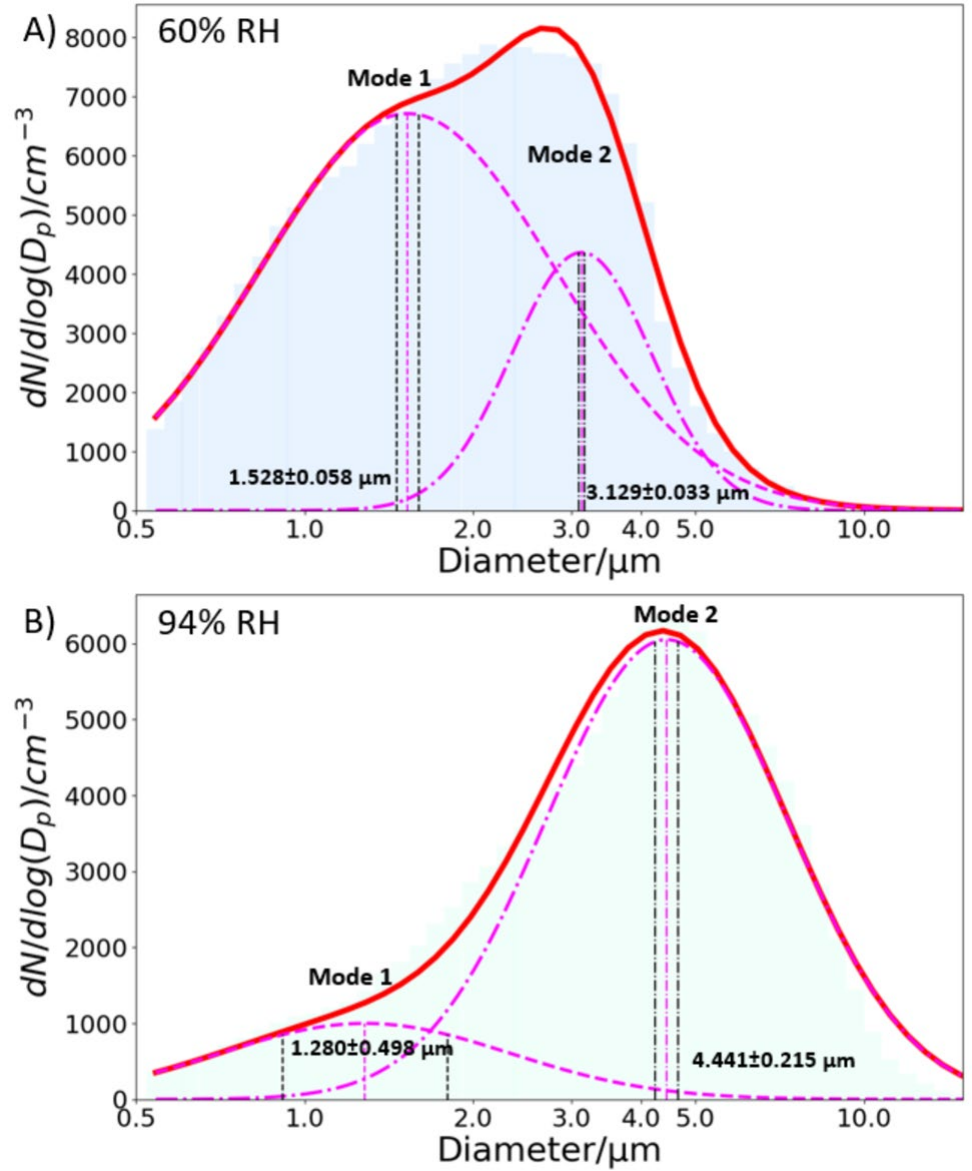
3% w/w NaCl CHAPS output for the first 3 stable seconds, with a bimodal distribution (pink) fitted using Python with (**A**) 60 ± 1% RH for APS_dry_, and (**B**) 94 ± 1% RH for APS_wet_. The vertical pink dashed lines represent the mean of the normal distribution, and the two black dotted lines on either size of the mean indicate the error range of the fitting.

An important observation from the spray development heatmaps is the dynamic nature of the aerosol plume. To facilitate a robust comparison of particle size distribution under varying RH conditions and formulations, we analyse and average the first 3 stable seconds of plume development, during which the size distribution remains stable and exhibits no significant changes. Figure 3 shows the measured size distribution using this approach. The clear reduction in size of the larger diameter mode (referred to as mode 2) from higher RH (median size = 4.441 ± 215 μm, Fig. 3B) to lower RH (median size = 3.129 ± 0.033 μm, Fig. 3A) indicates that a drier environment results in a decrease in particle size, as anticipated. Mode 2 is chosen for further comparisons because all particle sizes contributing to the mode remain within the detection limit of the APS even under drier conditions. As illustrated in Fig. 3, the first mode (mode 1) at 60% RH is not fully characterised and appears much flatter, leading to a larger error in the mean size estimate. The relative shape of the size distribution also changes between different RHs: at higher RH, the larger size mode is more pronounced than the smaller size mode, while at lower RH, the smaller mode has a similar particle concentration to the larger mode, causing the two modes to merge.

The CHAPS method allows a high RH (90% +) condition to be maintained in APS_wet_, while the RH in APS_dry_ can be adjusted using the Nafion dryer at room temperature (20°C). The measurements are typically taken when the APS_wet_ is maintained at around 95% RH to simulate the high humidity conditions in the lungs, and at 50–60% for APS_dry_ to represent ambient humidity conditions. Additionally, this RH range is used because the efflorescence RH of NaCl is around ~ 47% RH [27], below which NaCl droplets crystallize and lose their spherical shape. Consequently, particle size would require further interpretation, accounting for shape correction factors. The efflorescence RH for most tested ingredients, including mannitol and salbutamol sulphate, is lower than 60%, according to CK-EDB results (Figure S2).

The E-AIM rGFr of single droplet is calculated using Eq. 2, and the CHAPS rGFr is calculated as shown by Eq. 3.

2 $$rGFr=\frac{rGF\left({RH}_{high}\right)}{{rGF(RH}_{low})}$$

3 $${rGFr}_{plume}=\frac{{Mode2}_{{APS}_{wet}}}{{Mode2}_{{APS}_{dry}}}$$

Using the CHAPS size distributions in Fig. 3 as an example, the ${rGFr}_{plume}$ can be calculated as 4.441 ± 0.215 / 3.129 ± 0.033 = 1.419 ± 0.085. The uncertainty arises from the error in mode of the bimodal fittings, as illustrated in Fig. 3. The aerodynamic rGFr, converted from the E-AIM output, is 1.513 ± 0.153, with the uncertainty based on the RH sensor accuracy, which is ± 1% at RH values below 90% and increases to ± 1.7% at RH values above 90%. The agreement between the rGFr values from the E-AIM and CHAPS methods confirms the consistency between the two approaches.

**Fig. 4:**
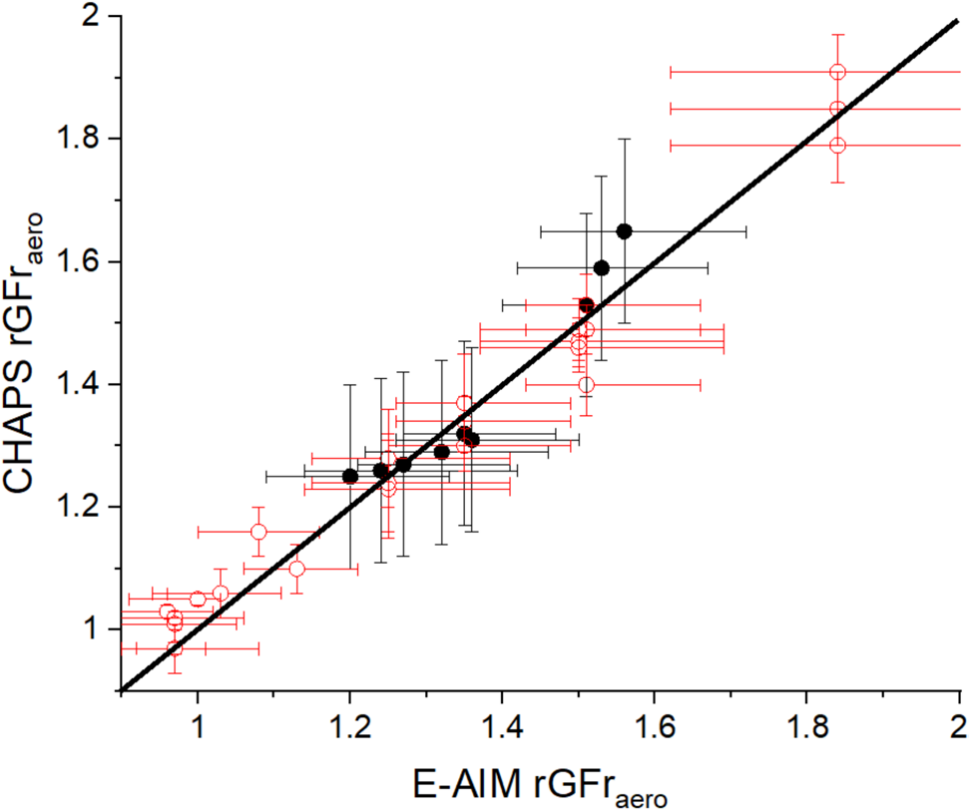
Comparison between the E-AIM rGFr_aero_ of aqueous NaCl aerosol and the CHAPS rGFr_aero_ measurements actuated from an Omron nebulizer (red open dots) and the Respimat® SMI (black closed dots). The black line has a gradient of 1. The error bar for the CHAPS rGFr_aero_ is based on the error range of the bimodal fitting (shown in Fig. 3) and the error bar for the E-AIM rGFr_aero_ is based on the uncertainty in the RH sensors.

Figure 4 compares that the aerodynamic rGFr (rGFr_aero_) of a 3% w/w sodium chloride aqueous solution (with water as the sole solvent, 0% ethanol) actuated from a Respimat® SMI, extracted from the CHAPS output, with the E-AIM predicted rGFr_aero_ across all the RH pairs. To compare the changes in plume size distribution actuated from Respimat® SMI (black closed dots), the RH in APS_wet_ is maintained at 94 ± 1.7%, while the RH condition in APS_dry_ ranges from 60 ± 1% to 85 ± 1%. The data points align closely with the 1:1 slope and the level of agreement is similar to that achieved in our previous work when using CHAPS to examine the hygroscopic growth of nebulised aqueous sodium chloride aerosol (red open dots).

**Fig. 5:**
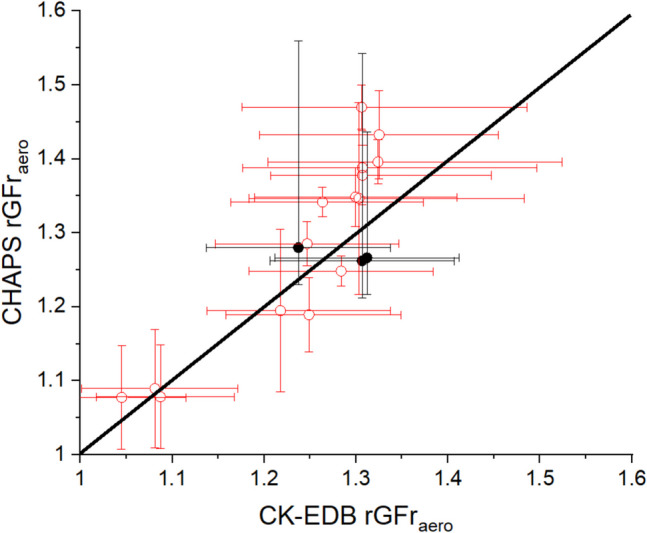
Comparison between the CK-EDB rGFr_aero_ and the CHAPS rGFr_aero_ actuated from Omron nebulizer (red open dots) and Respimat® SMI (black closed dots) using a of 3% w/w aqueous mannitol formulation. The black line has a gradient of 1.

We have determined the growth curve of mannitol experimentally using the CK-EDB technique and the single particle data used to estimate the rGFr_aero_ and compared with the CHAPS measurements in Fig. 5. The results indicate that the rGFr_aero_ values for aerosols actuated from Respimat® SMI align closely with those produced by the Omron nebulizer and with the single particle measurements. This suggests a consistent hygroscopic behaviour between mannitol aerosols delivered via nebulization and those delivered via the SMI and the alignment between plume and single droplet rGFr values validates the use of CHAPS for size distribution analysis. Both atomisation sources are consistent with predictions from the single particle measurements.

With a similar RH maintained for all the CHAPS APS_wet_ measurements (~ 95%), the absolute amount of aerosol delivered by the SMI during an actuation can be compared. Table II reports the average particle count and aerosol mass delivered over the stable 3 s in APS_wet_. The CHAPS method shows that there is not only good reproducibility for both NaCl and mannitol aerosol generation, but comparable masses are delivered per second. The minor deviation in particle count can be attributed to slight fluctuations in the RH during the experiments.

**Table II:** Reproducibility of Particle Count and Mass Delivered per Second by CHAPS APS_wet_ at 96 ± 2% RH for Sodium Chloride and Mannitol

95.0 ± 1.7% RH	Average Particle Count per second	Standard Deviation of Particle Count	Mass delivered per second (μg)	Standard Deviation of Mass
Sodium Chloride	19673.4	473.1	2.327	0.170
Mannitol	21203.8	267.9	2.477	0.132

### Influence of Ethanol on Aerosol Size Distribution

The higher vapor pressure and lower boiling point of ethanol compared to water result in a faster evaporation rate for ethanol as a solvent, leading to increased surface enrichment of involatile solutes due to a high Peclet number [28–30]. Gregson *et al*. [29] demonstrated that during the evaporation of aqueous-ethanol droplets into a humid environment, ethanol can fully evaporate while water can evaporate or condense depending on the RH. Indeed, for a droplet initially composed of just ethanol evaporating into a humid environment, the droplet can become pure water over a 1 s timescale driven by evaporative cooling. The presence of ethanol in inhalable formulations can influence initial and evolving aerosol droplet size distributions, residual particle shape, solubility, and stability, as well as the spray characteristics of the device [31, 32]. Patel *et al*. [33] conducted a double-blind study to confirm the safety of ethanolic solutions (up to 96% ethanol) administered via Respimat SMI in asthma patients. Therefore, optimizing ethanol content in formulations could provide a valuable approach to fine-tuning spray size distribution and enhancing therapeutic efficiency in soft mist inhalers (SMIs).

#### CHAPS and EDB Analysis of Ethanol and RH Effects on Mannitol and SS Aerosols

**Fig. 6:**
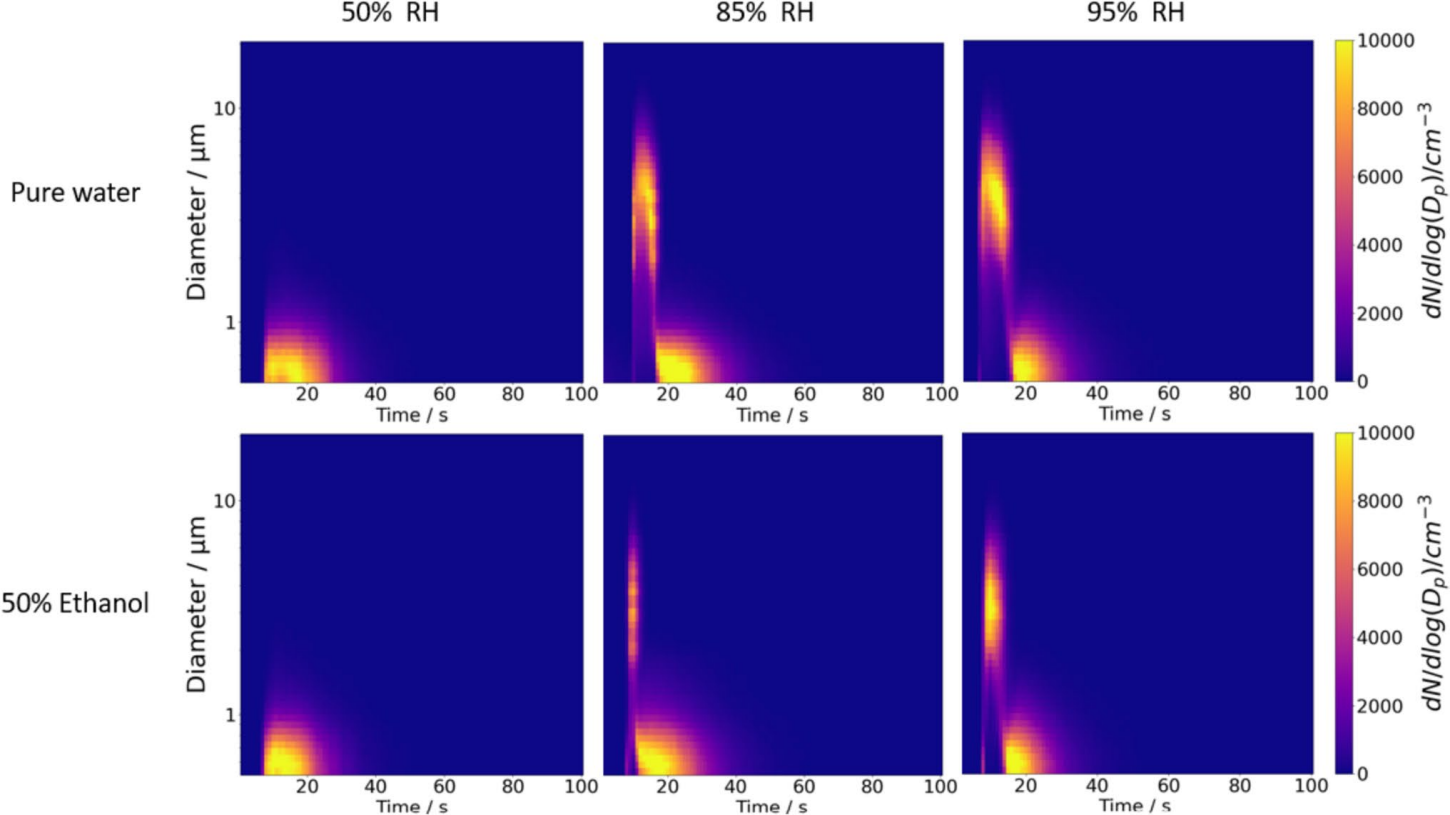
Plume development of 2% w/w mannitol actuated from the Respimat® SMI at 20°C at 50%, 85% and 95% RH dissolved in either pure water (0% v/v ethanol) or 50% v/v ethanol. The x-axis shows the time over the entire sampling period and the y-axis shows the particle size. The colour intensity represents the particle concentration

**Fig. 7:**
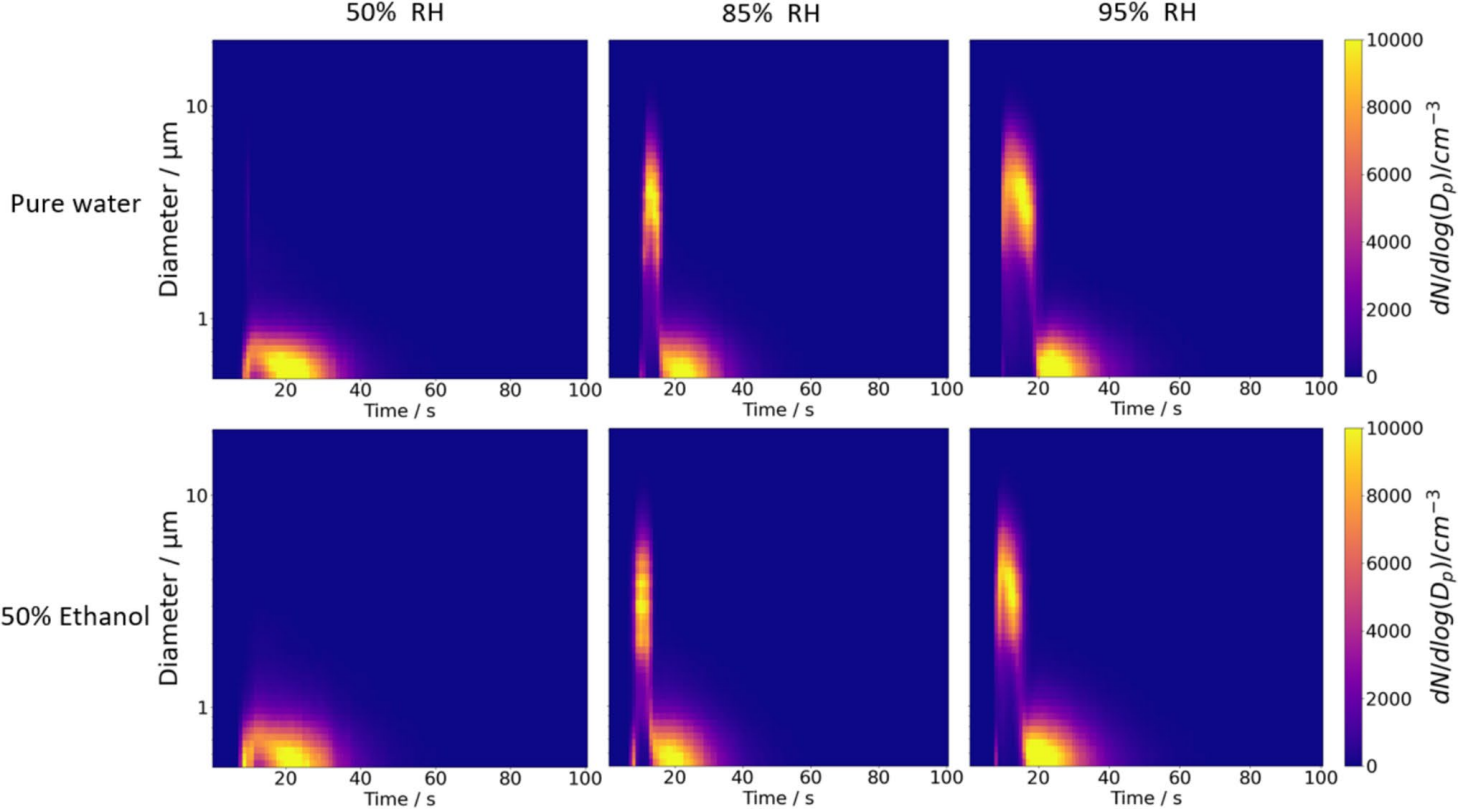
Plume development of 2% w/w salbutamol sulphate actuated from the Respimat® SMI at 20°C at 50%, 85% and 95% RH dissolved in either pure water (0% v/v ethanol) or 50% v/v ethanol. The x-axis shows the time over the entire sampling period and the y-axis shows the particle size. The colour intensity represents the particle concentration

In this study, we employ the CHAPS method to examine the influence of ethanol on the actuated particle size distribution, while also exploring the effect of RH. Furthermore, we evaluate the consistency of ethanol’s impact across a range of ethanol–water solvent-based solutions, from pure water (0% v/v ethanol) to 75% v/v ethanol by volume in 25% increments. The CK-EDB measurements indicate that 2% w/w aqueous mannitol droplets (pure water as solvent) crystallize upon reaching equilibrium at 50% RH (Figure S2A), whereas aqueous salbutamol sulphate (SS) droplets crystallize below 55% RH upon equilibrium (Figure S2B). Since the increase in the rGF curve gradient remains relatively flat until humidity exceeds 90%, we maintain 95% RH in APS_wet_ to simulate the high RH environment in the lung, and use 85% RH in APS_dry_ to observe size distribution changes due to RH variations while preventing crystallization. Figures 6 and 7 depict the changes in size distribution captured by CHAPS of the SMI actuations using different solvent composition, specifically 0% v/v ethanol (pure water) and 50% v/v ethanol for mannitol and SS, respectively. The dry RH conditions were set at 50 ± 1%, 85 ± 1% and 95 ± 1.7%.

Both the mannitol (Fig. 6) and the SS (Fig. 7) plumes show few particles larger than 2 μm at 50 ± 1% RH when pure water is used as the solvent. This observation aligns with the phase function data (Figure S2) obtained from the CK-EDB, which show the phases of both SS and mannitol droplets transition from homogeneous droplets at the point of actuation to droplets with inclusions, eventually crystallizing as they equilibrating with the surrounding RH (~ 50%). The influence of increasing ethanol content as a volatile solvent, particularly at 85% and 95% RH on both mannitol and SS formulations, is further examined in the following sections.

#### The Impact of Ethanol Concentration on the Particle Size Distribution of Aqueous-Ethanol-Mannitol Aerosol

**Fig. 8:**
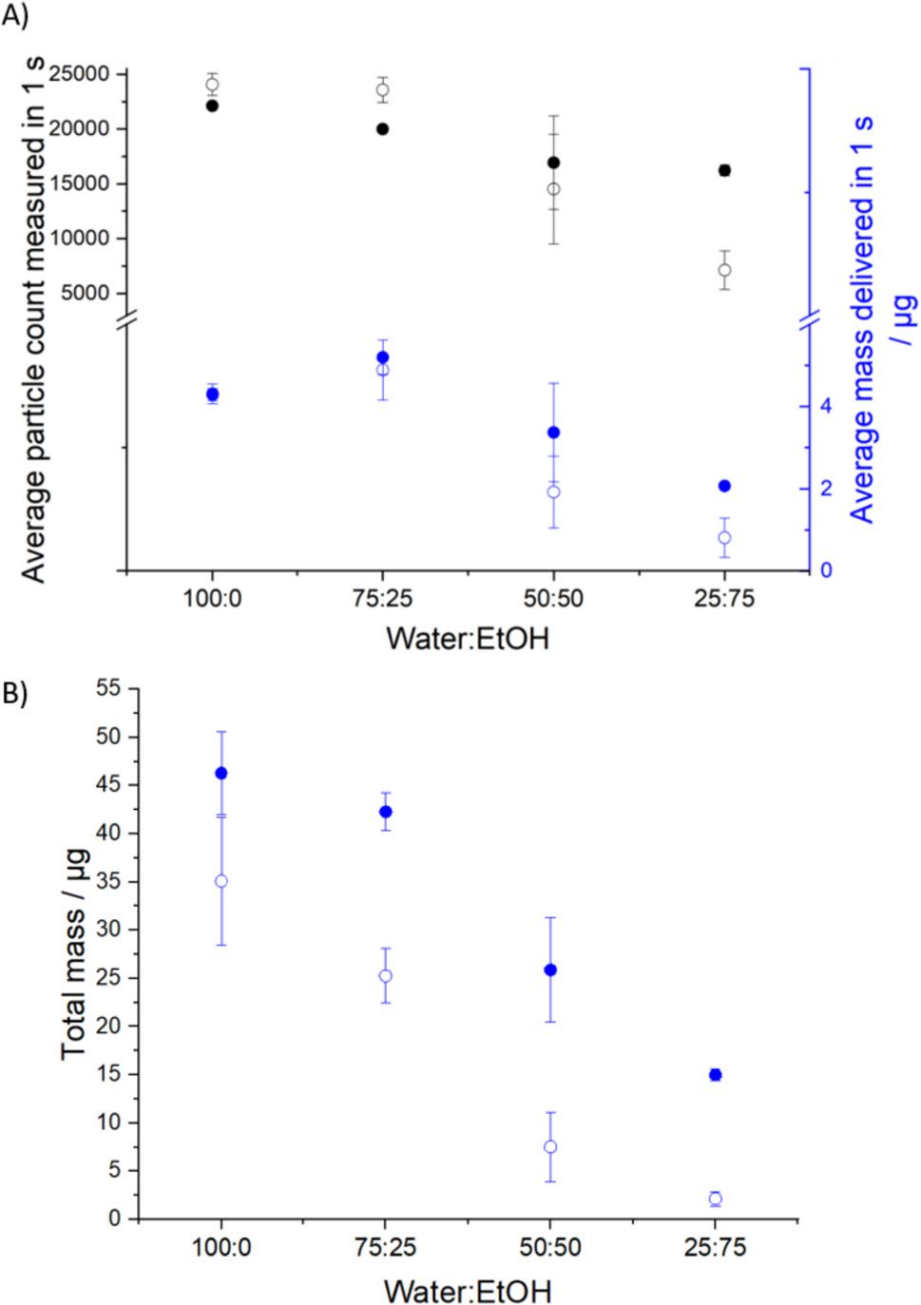
2% w/w mannitol CHAPS results (*n* = 4 for each ratio) with a range of water to ethanol ratios at low and high RH conditions (*n* = 3). APS_dry_ (85 ± 1% RH) is presented using open circles, while APS_wet_ (95 ± 1.7% RH) is presented using filled circles. (**A**) Black symbols show the average particle number concentration over the stable 3 s. The blue symbols show the average mass delivered in 1 s averaged over the stable 3 s. (**B**) The cumulative mass of mannitol over the entire actuation.

The impacts of increasing ethanol concentration in the 2% w/w mannitol formulation on both particle number concentration and mass are illustrated in Figs. 6B and 8A. Figure 8A presents the average particle count and mass delivered per second over the first 3 stable seconds, with ethanol content varying from 0% v/v (pure water as solvent) up to 75% v/v in 25% increments. The upper limit of 75% v/v ethanol ensures complete solute dissolution without forming a suspension of mannitol. The RHs for APS_dry_ and APS_wet_ are maintained at 85% and 95%, respectively. A continuous decline in particle number concentration is observed in APS_wet_ as ethanol content rises; for APS_dry_, a more pronounced decrease occurs above 25% v/v ethanol. The average mass trend with RH mirrors this behaviour, with no significant change up to 25% ethanol but a significant decrease at higher ethanol concentrations (Fig. 8A). The total aerosol mass delivered can be determined by summing the cumulative mass per second throughout the entire sampling duration, as measured by CHAPS (Fig. 8B). The difference in mass between APS_dry_ and APS_wet_ becomes increasingly significant, with a noticeable difference emerging even between pure water and 25% v/v ethanol compositions. The lower mass in the dry-channel reflects the much lower water content in the aerosol at lower RHs, increasing from a $\frac{{APS}_{wet}}{{APS}_{dry}}$ ratio 1.32 ± 0.46 to 7.04 ± 4.66 as the ethanol percentage increases from 0 to 75% (Fig. 8). An aerosol that starts from the SMI with lower water content, ends up with lower water content and this is particularly evident at the lowest RH.

**Fig. 9:**
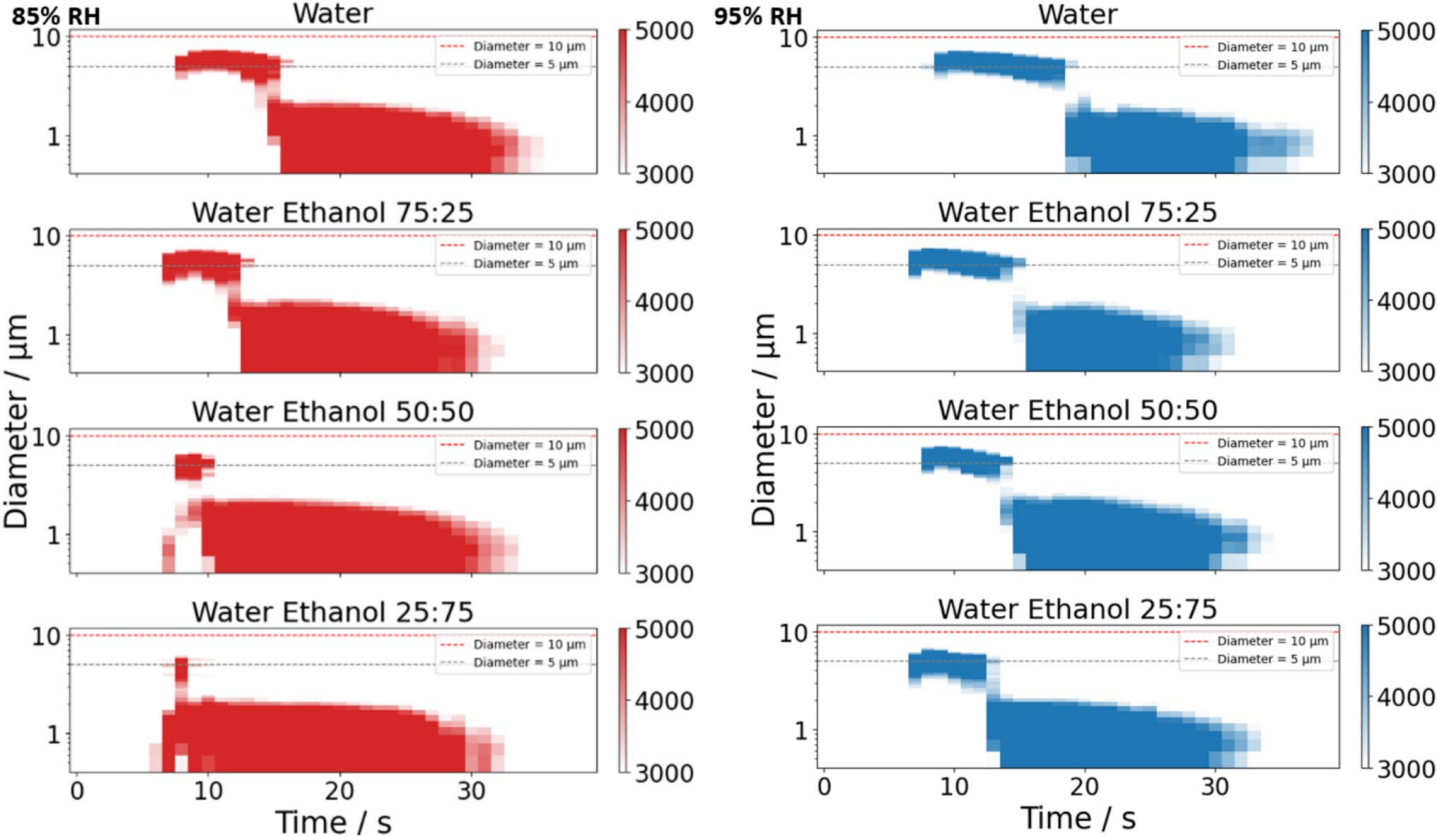
Plume development obtained from the CHAPS size distribution data with 1 s time resolution of 2% w/w mannitol with 0 – 75% v/v ethanol content at 85% RH (left column) and 95% RH (right column). The colour bar represents the particle number concentrations (dN / dlog(D_p_) / cm^−3^).

The discrepancy between the total mass for pure water (0% ethanol) and 25% ethanol content observed in Fig. 8B, which is not reflected in the average mass depicted in Fig. 8A, can be attributed to the plume dynamics illustrated in Fig. 9. The subplots in Fig. 9 present plume development plots (similar to Fig. 2). Each sample recording begins with ~ 10 s of background readings, followed by actuation. The colour gradation is specifically chosen to focus on the change in size distribution near the peak of the number concentration distribution. As the ethanol concentration increases at both RHs, the presence of larger particles around 5 μm (indicated by the red line in Fig. 9) is less sustained, with them present over a shorter time window. Concentrations of particles with sizes below 5 μm diameter (as indicated by the grey line) are more consistent and are less affected by variations in ethanol concentration. In all cases, the reduction in the duration of the larger particle production leads to a significant decrease in mass (Figure S4). This reduction is most significant at low RH.

An example of the size distribution at different time points, average over 3-s intervals and at 85% RH, is shown in Figure S3. The actuation from the SMI initially produces a peak around 5 μm diameter (Figure S3, 12–14 s). After 8 more seconds (Figure S3, 20–22 s), the 5 μm peak diminishes in magnitude, while a new peak emerges below 1 μm. By the end of plume development (Figure S3, 30–32 s), only the submicron peak remains, likely resulting from resuspension of deposited liquid within the CHAPS system. Increasing the ethanol concentration shortens the duration of the initial peak observed at ~ 5 μm (Figure S3, 12–14 s) due to the higher evaporation rate.

#### The Impact of Ethanol Concentration on the Particle Size Distribution of Aqueous-Ethanol-Salbutamol Sulphate Aerosol

Salbutamol sulphate (SS) is a short-acting β2-adrenergic receptor agonist that acts on smooth muscle in the respiratory system to induce bronchodilation [34]. It is widely used in the treatment of asthma and other respiratory disorders [34–36]. Although inhaled SS formulations typically do not exceed a concentration of 1 mg/mL (0.1% w/w) in clinical practice [37], a higher concentration (2% w/w) is employed in this study to facilitate comparison with mannitol results and accommodate the requirements of methods like CK-EDB.

**Fig. 10:**
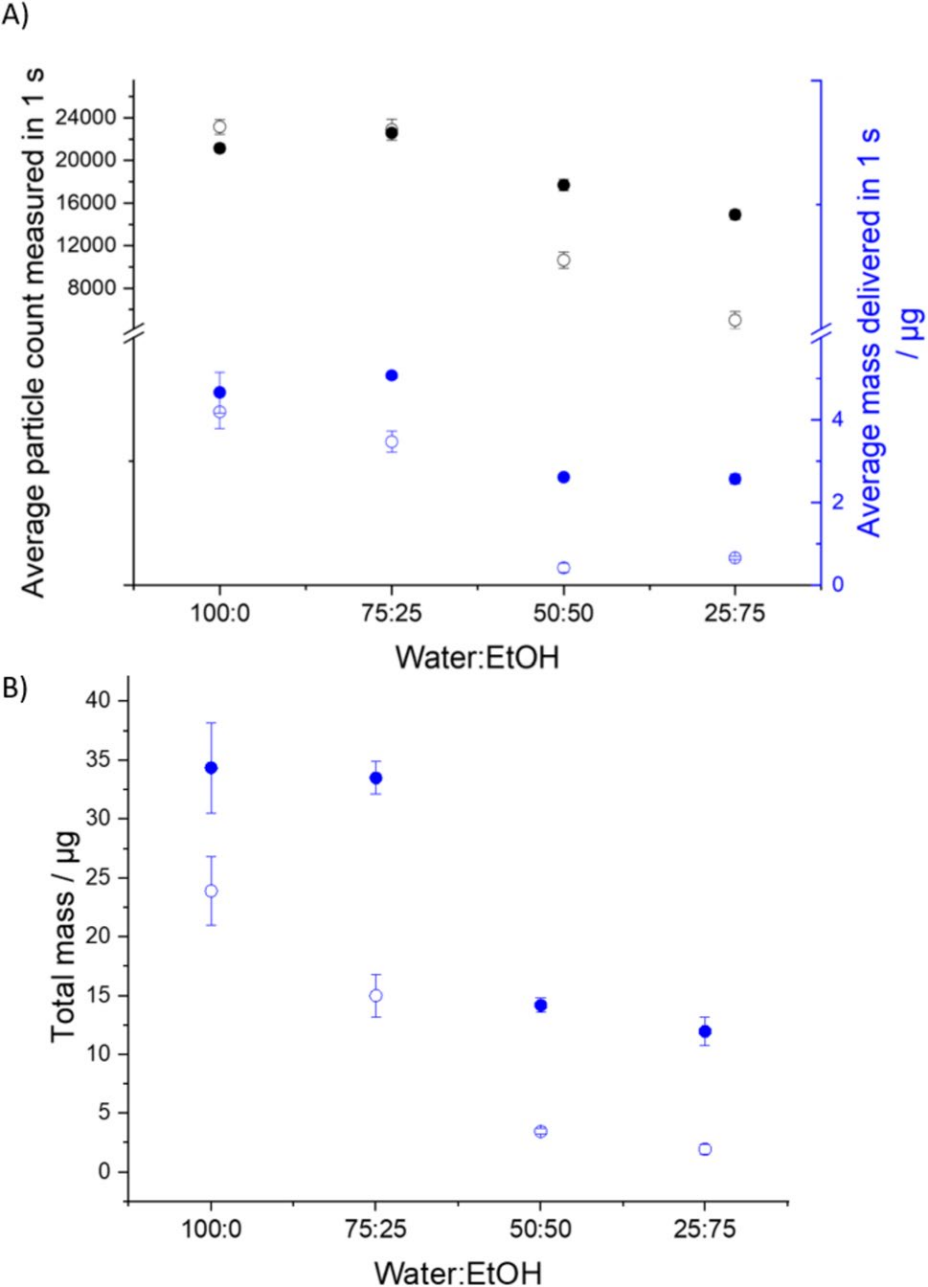
2% w/w SS CHAPS results (*n* = 4 for each ratio) with a range of water to ethanol ratios at low and high RH conditions (*n* = 3). APS_dry_ (85 ± 1% RH) is presented using opened circles, while APS_wet_ (95 ± 1.7% RH) is presented using filled circles. (**A**) Black symbols show the average particle concentration over the stable 3 s. The blue symbols show the average mass over the stable 3 s. (**B**) The cumulative mass of mannitol over the entire sampling period.

The effect of increasing ethanol concentration on SS formulations mirrors the patterns observed with mannitol. As shown in Fig. 10A, both APS_wet_ (95 ± 1.7% RH) and APS_dry_ (85 ± 1% RH) exhibit a decrease in average particle concentration and mass during the first three stable seconds as ethanol content increases from 25% v/v to 75% v/v. While pure water (0% v/v ethanol) and 25% v/v ethanol solutions maintain comparable levels of average mass and particle count, a significant difference in total mass (Fig. 10B) is evident in APS_dry_ between 0% v/v and 25% v/v ethanol concentration in the solvent. The most pronounced reduction in mass for both APSs occurs between 25% v/v and 50% v/v ethanol. These results again reflect the lower water content at lower RH and the higher water content in the aerosol when the atomised solution is initially dominated by water.

**Fig. 11:**
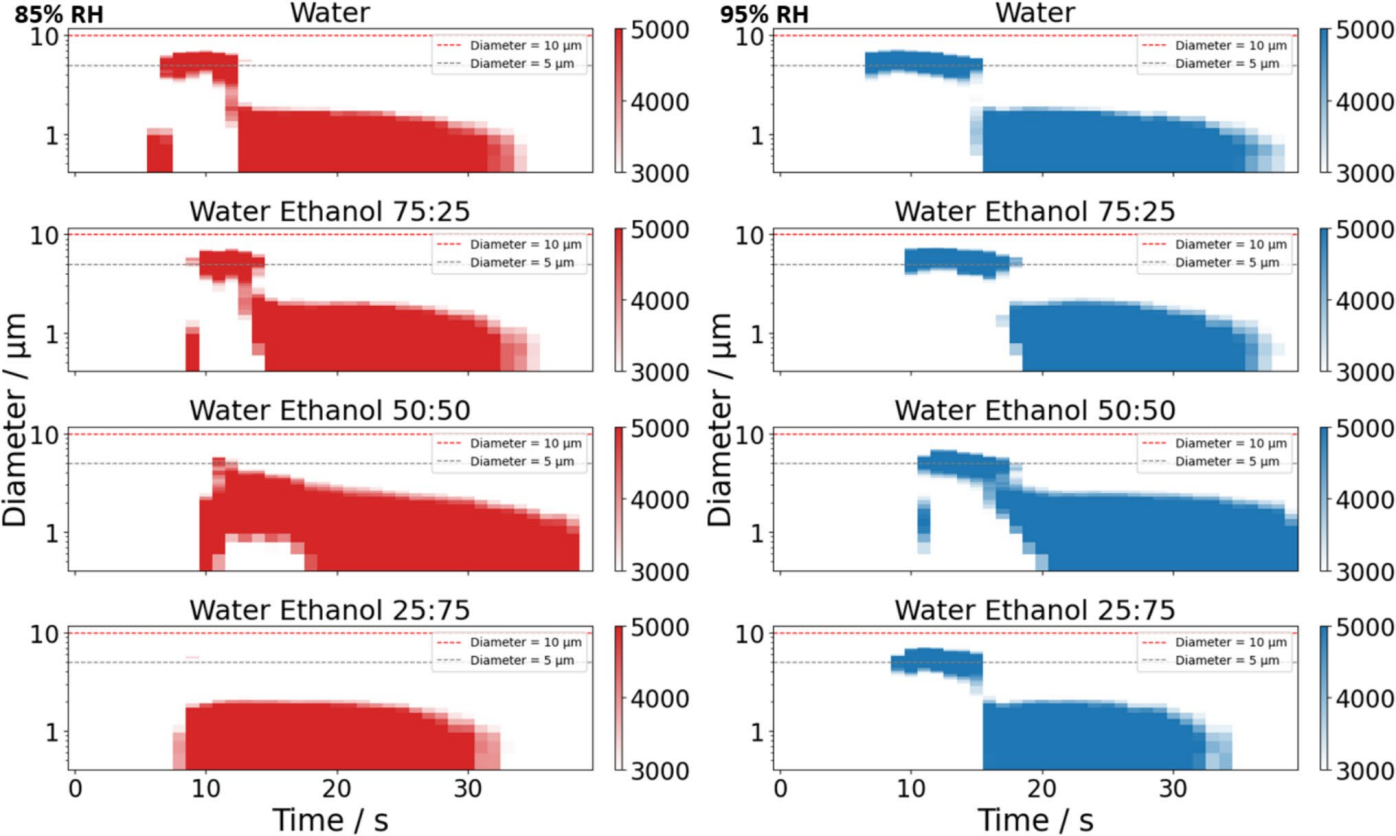
Plume development obtained from the CHAPS size distribution data with 1 s time resolution of 2% w/w salbutamol sulphate with 0 – 75% v/v ethanol content at 85% RH (left column) and 95% RH (right column). The colour bar represents the particle number concentrations (dN / dlog(D_p_) / cm^−3^).

The influence of increasing ethanol concentration on SS formulations is further illustrated in Fig. 11, which presents the plume development profiles. At 85% RH, the presence of ~ 5 μm diameter aerosols (Fig. 11) becomes shorter in duration as ethanol content increases from 0% v/v to 75% v/v. Similarly, the particle mass (Figure S5) decreases with the rising ethanol ratio. When the RH is increased to 95%, the larger particles (~ 5 μm diameter) are sustained for a longer period, though the effect of increasing ethanol concentration mirrors the trend observed at 85% RH. At 95% RH, the differences in particle count and mass between pure water solvent (0% v/v ethanol) and 25% v/v ethanol are minimal. However, once the ethanol concentration surpasses 50% v/v, a significant reduction in both particle mass and count is apparent. This reduction is especially pronounced at the lower RH of 85%, which is reflected in the total mass (Fig. 10B) becoming almost imperceptible due to the scarcity of larger particles.

Despite salbutamol sulphate being a hygroscopic compound that is freely soluble in water, and mannitol being characterized by low hygroscopicity, the impact of ethanol on particle count and mass is consistent and substantial across both substances. As ethanol content increases, both particle count and mass decrease, with the most significant reduction in particle size occurring between 25% v/v and 50% v/v ethanol content. This suggests that the ethanol-induced reduction in particle size is a robust phenomenon, irrespective of the hygroscopic nature of the solute.

### Mechanistic Interpretation: Kinetics of Ethanol and Water Evaporation

The interplay between ethanol concentration, RH, and droplet size can be understood through considering the evaporation kinetics of the multicomponent and multi-volatile aerosol. There is a complex coupling between particles in the evolving cloud and an interplay between the mass and heat transfer at an individual particle level. However, as a first step to analysing this complexity, the rate of evaporation of a volatile component from an aerosol droplet is governed by its vapor pressure, diffusivity, and the surrounding RH. Given the significant difference in vapor pressures between ethanol (5.95 kPa) [38] and water (2.34 kPa) [39], ethanol exhibits a much faster evaporation rate compared to water under ambient conditions. This differential evaporation behaviour leads to a rapid depletion of ethanol from the droplet surface, altering the solute concentration and ultimately influencing the droplet size distribution.

Assuming steady evaporation conditions are achieved promptly, the characteristic evaporation timescale for ethanol and water in a binary droplet system can be estimated using the squared-radius law, given by $${R}_{p}^{2}={R}_{p,0}^{2}-S(t-{t}_{0})$$, where $$t$$ is time, $${R}_{p}$$ is the droplet radius at $$t$$, $${R}_{p,0}$$ is the droplet radius at time zero ($${t}_{0})$$, and $$S$$ represents the evaporation rate coefficient. The expression for $$S$$ is derived as:

$$S=\frac{2{D}_{g}{M}_{i}{p}_{i}^{0}(T)}{{\rho }_{i}RT}$$

where ${D}_{g}$ is the diffusivity of $i$, ${M}_{i}$ is the molar mass of $i$, ${p}_{i}^{0}(T)$ is the saturated vapour pressure of $i$ at temperature $T$, ${\rho }_{i}$ is the density of $i$ in liquid phase, R is the universal gas constant (8.314 J/(mol$\bullet K)$) and $T$ is the absolute temperature.

Since $S$ has units of ${s}^{-1}$, a characteristic timescale for complete evaporation can be estimated as:

$$t=\frac{{R}_{p,0}^{2}}{S}$$

Substituting the expression for $S$:

$$t=\frac{{R}_{p,0}^{2}{\rho }_{i}RT}{2{D}_{g}{M}_{i}{p}_{i}^{0}(T)}$$

Including a reduction in the effective vapour pressure of the evaporating component by the saturation fraction $Sat$ in the gas phase reflects the reduction in the diffusional vapour pressure gradient from the droplet surface to the surrounding vapour, leading to:

$$t=\frac{{R}_{p,0}^{2}{\rho }_{i}RT}{2{D}_{g}{M}_{i}{p}_{i}^{0}(T)(1-Sat)}$$

The consequence of this differential evaporation is that the droplet evaporation timescale is reduced at increasing ethanol concentration (mole fraction) due to the higher vapor pressure for the ethanol component, compared with water. This results in a faster depletion of droplet volume. This effect is evident in the experimental data (Figs. 7 and 9), where increasing ethanol content leads to a reduction in both the particle number concentration (above the size detection threshold) and mass. The transition from water-dominated to ethanol-dominated evaporation kinetics is also observed in the time-resolved plume development, where larger particles persist for shorter durations as ethanol concentration increases. As already stated, the complex coupling between particles in the evolving cloud, the size dependent evaporation rates, the dependence on the RH of the flow and the changes in volatile composition in the gas phase all impact on the dynamical changes in size distribution and a full analysis of this is beyond what can be achieved in the current study.

## Conclusion

Particles generated from a SMI device undergo interaction and equilibration with the surrounding RH conditions. The growth factor of the aerosol plume, as measured by the CHAPS instrument, demonstrates a high degree of concordance with the growth factors derived from CK-EDB measurements of single droplets. This strong agreement suggests that the size distribution of SMI-generated aerosols can be reliably modelled and predicted based on single droplet data.

This study provides the first *in situ* characterization of both the particle number and mass of particles generated during device actuation, alongside a comparative analysis of size distributions at two distinct relative humidities and an evaluation of actuation repeatability. Unlike previous studies that primarily measure residual particles (e.g., using NGIs), our approach delivers high time-resolution data, enabling a detailed understanding of the evolving aerodynamic particle size distributions under controlled RH conditions.

Increasing the ethanol content in the formulation has been shown to consistently reduce both particle size and mass. While potential health implications, such as respiratory tract irritation, must be carefully considered, strategically adjusting the ethanol content presents a valuable approach for optimizing particle size distribution. A higher ethanol percentage can enhance the efficiency of inhaled therapies by accelerating evaporation, ultimately resulting in a higher fine particle fraction. Therefore, it is essential to strike a balance between maximizing therapeutic efficacy and minimizing adverse effects.

## Supplementary Information

Below is the link to the electronic supplementary material.

Supplementary file1 (DOCX 589 KB)

## Data Availability

Data are available at the University of Bristol data repository, data.bris, at 10.5523/bris.wkc0gutavnil2atq41luu6s2e.
