# Supplementary material for: Characterizing the Influence of Relative Humidity and Ethanol Content on the Dynamic Size Distributions of Aerosols Generated from a Soft Mist Inhaler
Source: Pharm Res. 2025 Apr 1;42(4):651–63. doi: 10.1007/s11095-025-03851-1 (PMC12055633; doi:10.1007/s11095-025-03851-1)
Supplement: Supplementary file 1 — Supplementary file1 (DOCX 589 KB) [file 11095_2025_3851_MOESM1_ESM.docx]

Characterizing the Effect of Relative Humidity and Ethanol Content on Aerosols Generated from a Soft Mist Inhaler - Supplementary

Yiliang Lance Jiang^1^, Jose R. Ruiz^2^, Richard Friend^2^, Jonathan P. Reid^1,*^

1. University of Bristol, School of Chemistry, Cantock’s Cl, Bristol, BS8 1TS, United Kingdom
2. Chiesi Ltd., 1 Bath Road Industrial Estate, Bath Rd, Chippenham, SN14 0AB, United Kingdom

^*^ - Corresponding author: j.p.reid@bristol.ac.uk


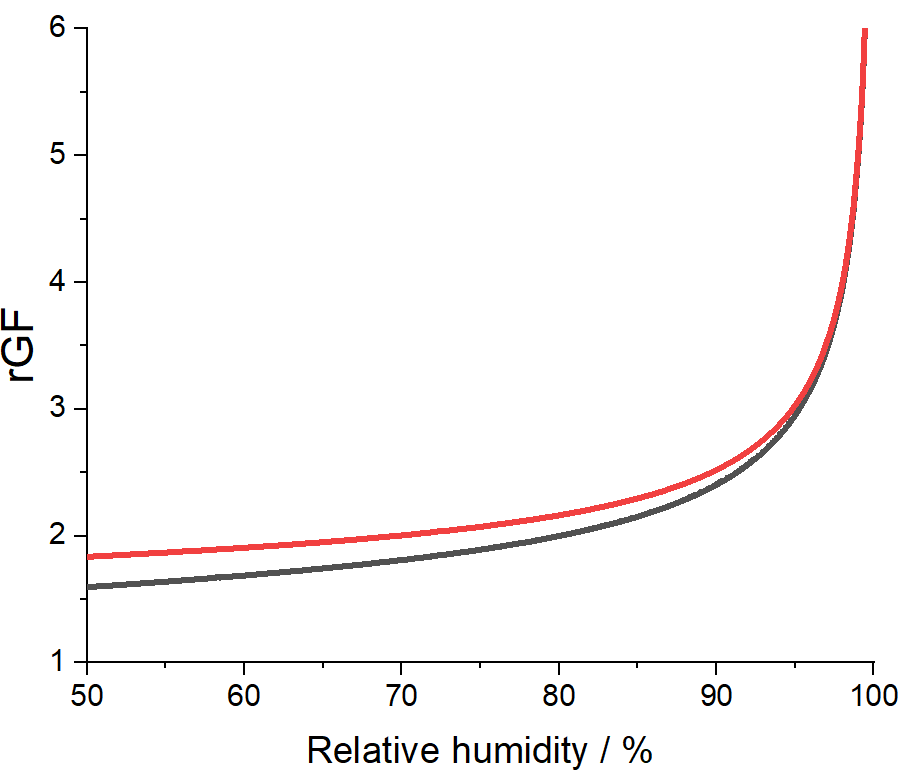


Figure S1 – Geometric rGF curve (black line) for NaCl derived from CK-EDB data and the aerodynamic rGF curve (red line) incorporating AIOMFAC output and density parameterization.


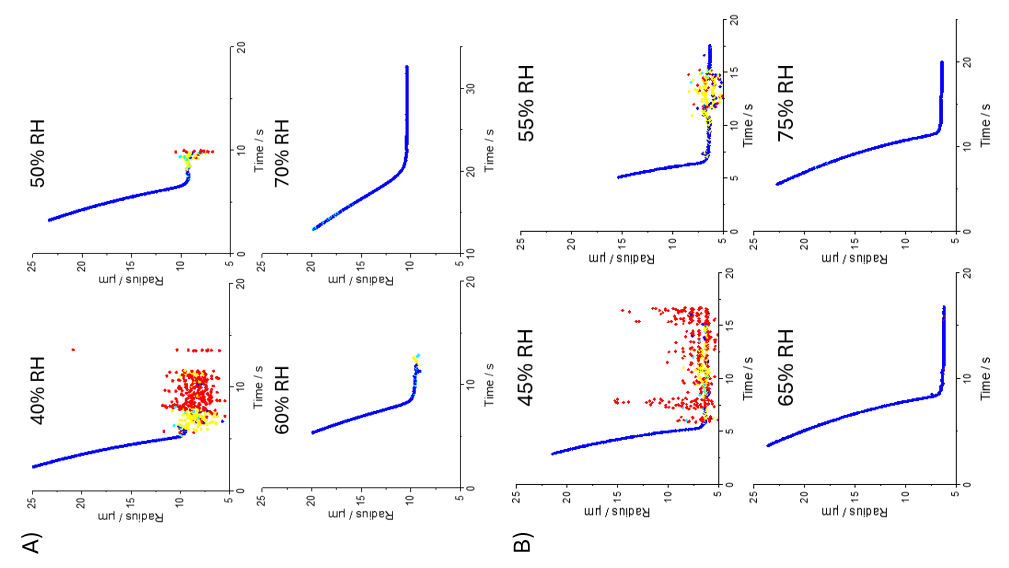


Figure S2 – Phase function data obtained from CK-EDB with A) 2% w/w mannitol in pure water, and B) 2% w/w salbutamol sulphate in pure water. The colour indicates particle type: blue is homogeneous, yellow is inclusions and red is crystallized.


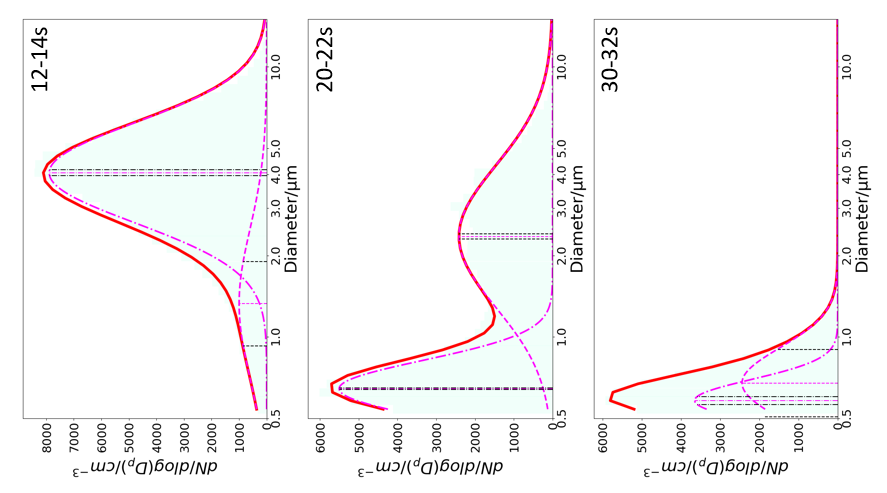


Figure S3 – 2% w/w mannitol (0% ethanol content) TAPS size distribution at different time points during plume development at 85% RH.


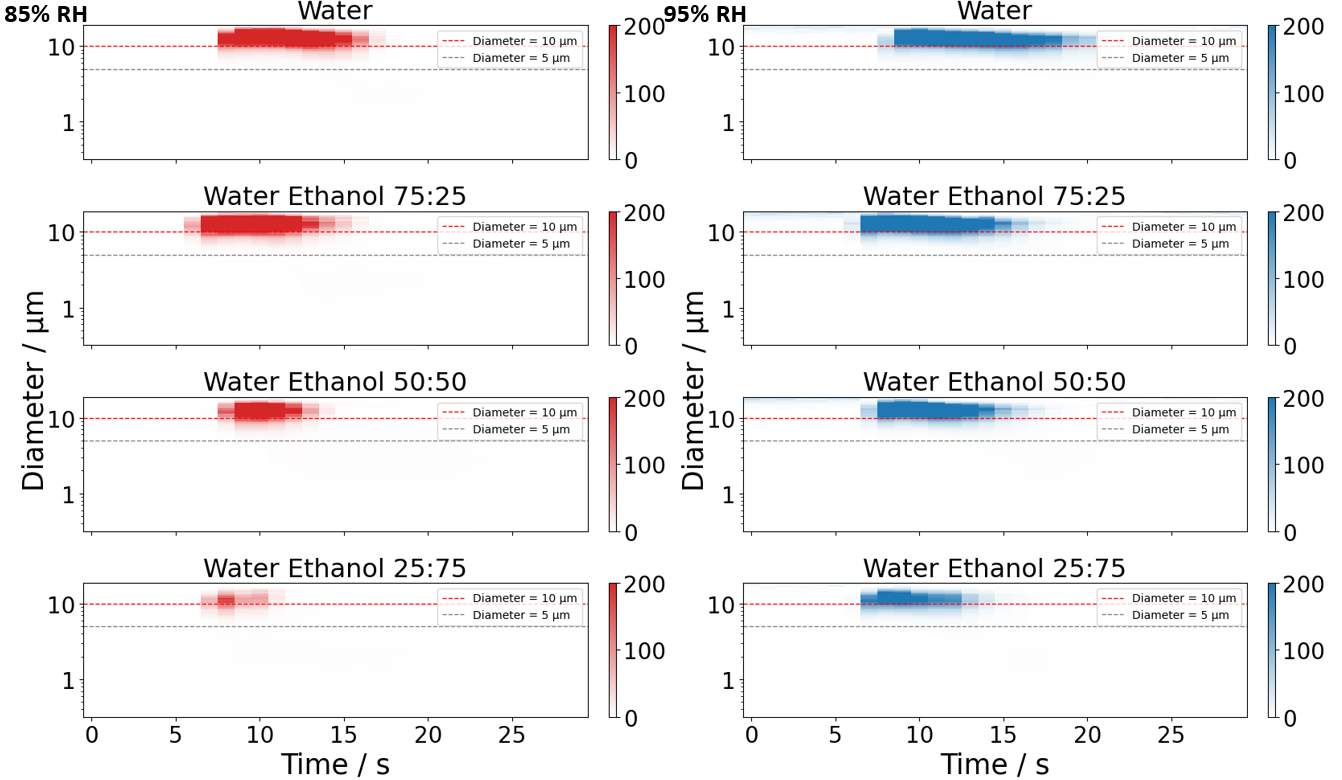


Figure S4 – Plume development obtained from the TAPS size distribution data with 1 second time resolution of 2% w/w mannitol with 0 – 75% v/v ethanol content at 85% RH (left column) and 95% RH (right column). The colour bar represents the mass concentrations (dM / dlog(D_p_) / cm^-3^).


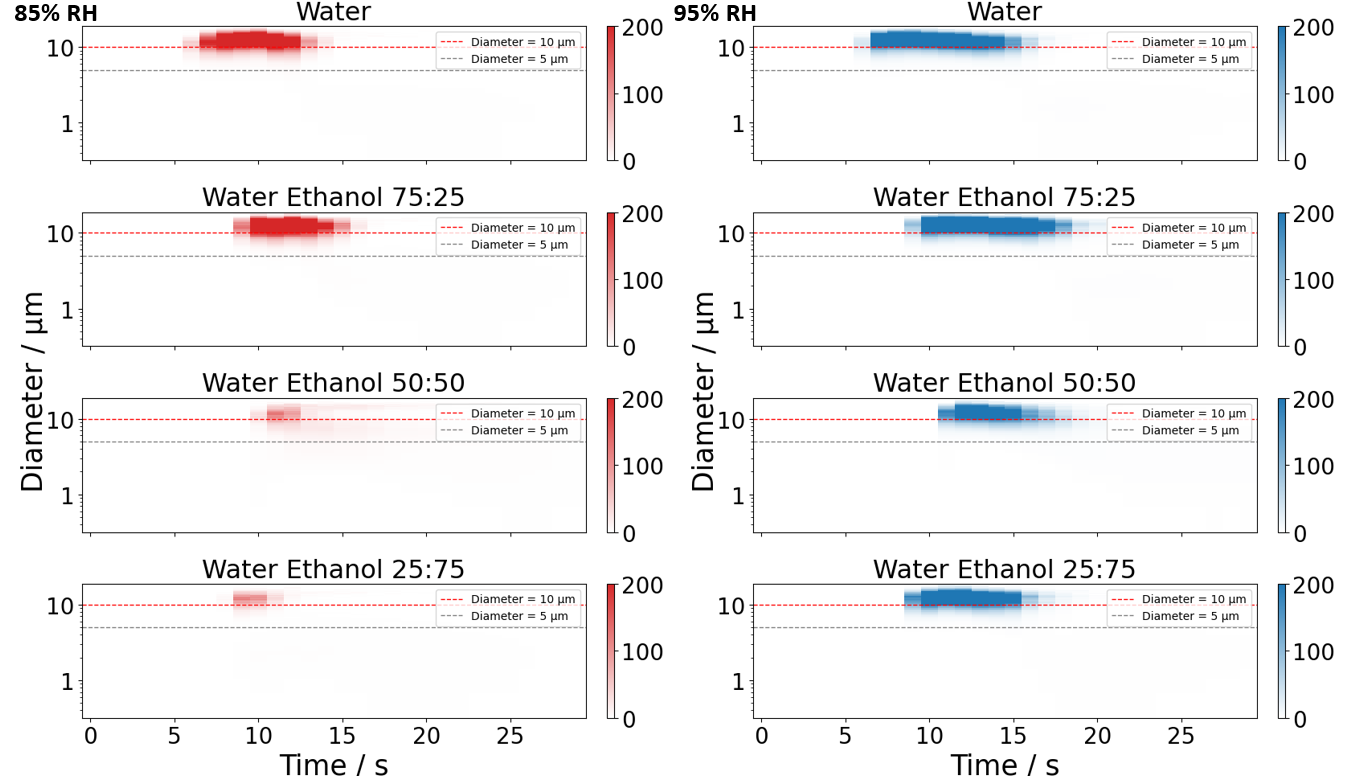


Figure S5 - Plume development obtained from the TAPS size distribution data with 1 second time resolution of 2% w/w salbutamol sulphate with 0 – 75% v/v ethanol content at 85% RH (left column) and 95% RH (right column). The colour bar represents the mass concentrations (dM / dlog(D_p_) / cm^-3^).
